# Ataxia-telangiectasia paradoxes: spotlight on post-zygotic chromosome instability in the brain and its contribution to neurodegeneration pathways

**DOI:** 10.1186/1750-1326-8-S1-P51

**Published:** 2013-09-13

**Authors:** Yuri Yurov, Svetlana Vorsanova, Thomas Liehr, Ivan Yurov

**Affiliations:** 1 Mental Health Research Center, Russian Academy of Medical Sciences, Moscow, Russia; 2 Institute of Paediatrics and Paediatric Surgery, Ministry of Health, Moscow, Russia; 3 Moscow City University of Psychology and Education, Moscow, Russia; 4 Institute of Human Genetics, Jena, Germany; 5 Department of Medical Genetics, Russian Medical Academy of Postgraduate Education, Moscow, Russia

## Background

Ataxia-telangiectasia is a syndrome of chromosome instability (CIN) featured by progressive neurodegeneration affecting the cerebellumin in contrast to other brain areas. Previously, progressive neuronal death in AT was hypothesized to be driven by increased post-zygotic CIN in the cerebellum [1-3]. To assess the possible involvement of neural CIN in neurodegenerative pathways we have analyzed CIN in AT brain.

## Materials and methods

CIN was evaluated by high-resolution single cell (immuno-) FISH techniques in 7 AT and 7 control samples. Bioinformatics analyses of neurodegeneration pathways were done as described earlier [4].

## Results

Global aneuploidization affecting 20% of neurons and 80% of glial cells in the prefrontal cortex and cerebellum was observed. CIN in the cerebellum has affected specific chromosomes (7, 14, and X) similarly the immune system. Paradoxically, dramatic and age-dependent increase in the pathological level of CIN did not result in accelerated neurodegeneration in the brain of patients, but was associated with increased lifespan. Bioinformatics analyses have shown that the SMAD pathway is implicated in neurodegeneration in addition to genome integrity maintenance, somatic V(D)J recombination and DNA damage response in neural cells.

## Conclusions

To explain paradoxes we hypothesized that progressive neurodegeneration in the cerebellum can be associated with activation of adult neurogenesis in the diseased cerebellum and/or activation and proliferation of microglia - the phagocyte system of the brain. Neurodegeneration pathways in the AT brain are likely to be applicable to disorders exhibiting neurogeneration in specific brain areas.
